# The Variability for the Biochemical Indicators at the Winter Wheat Assortment and Identifying the Sources with a High Antioxidant Activity

**DOI:** 10.3390/plants10112443

**Published:** 2021-11-12

**Authors:** Ramona Aida Paunescu, Elena Bonciu, Elena Rosculete, Gabriela Paunescu, Catalin Aurelian Rosculete, Cristina Babeanu

**Affiliations:** 1 Syngenta Agro Romania, 73-81 Bucuresti-Ploiesti Street, 013685 Bucharest, Romania; aida.paunescu@yahoo.com; 2 Department of Agricultural and Forestry Technology, Faculty of Agronomy, University of Craiova, 13 A.I. Cuza Street, 200585 Craiova, Romania; catalin_rosculete@yahoo.com; 3 Department of Land Measurement, Management, Mechanization, Faculty of Agronomy, University of Craiova, 13 A.I. Cuza Street, 200585 Craiova, Romania; 4 SCDA Caracal, University of Craiova, 106 Vasile Alecsandri Street, 235200 Caracal, Romania; paunescucraiova@yahoo.com; 5 Department of Chemistry, Faculty of Sciences, University of Craiova, 13 A.I. Cuza Street, 200585 Craiova, Romania; cbabeanu@yahoo.com

**Keywords:** wheat, peroxidase, ascorbate peroxidase, catalase, yield index

## Abstract

This study presents the variability of some biochemical indicators in the winter wheat assortments tested in south-western Oltenia (Romania) and identification of the sources showing a high antioxidant activity. The peroxidase activity has intensified as the stress induced by treatment with PEG of different concentrations and in different doses increased. Regarding the peroxidase content, among the varieties treated with PEG 10,000 25%, the majority of the Romanian varieties tested showed higher values of the PEG/control treatment ratio, which suggests tolerance to drought. In reverse, the activity of ascorbate peroxidase is lower in tolerant varieties. The varieties with a subunit report have been noted. Among them are the Izvor variety, known as the drought-tolerant variety, as well as other Romanian varieties: Alex, Delabrad, Lovrin 34, etc. An increased activity of catalase was present in most varieties, so there is the possibility of drought tolerance. Among the varieties highlighted are Romanian varieties (Dropia, Trivale, Nikifor, etc.) but also foreign varieties (Kristina, GH Hattyu, Karlygash, etc.). However, the correlation between yield index in the limited assortment and the antioxidant enzyme content ratios between PEG and control treatments does not exist, suggesting that none of these biochemical indicators are a selective indicator for drought tolerance under the experimental condition.

## 1. Introduction

Wheat is a cheap source of essential amino acids (which are not synthesized in the body), good quality minerals, vitamins, and vital dietary fibres to the human diet [1]. Besides this, it is also considered a natural source of both enzymatic and non-enzymatic antioxidants [2]. The enzymatic antioxidants include superoxide dismutase (SOD), glutathione reductase (GR), and ascorbate peroxidase (APX), catalase (CAT), and peroxidase (POD), while non-enzymatic antioxidants include vitamin C (tocopherols and tocotrienols), vitamin E, and carotenoids [3].

Drought tolerance is a complex trait that refers to the degree to which a plant is adapted to arid or drought conditions that lead to different morphological and physiological changes. Adaptation processes to drought stress conditions involve the genetics at different molecular, physiological, biochemical, and biological levels and processes [4,5].

Under drought conditions, oxidative degradation products occur at the cellular level, leading to oxidative stress. Numerous experiments on the study of wheat drought resistance showed cell-based induction of enzyme oxidative stress protection systems [6,7,8]. A drought-tolerant genotype had the highest activity of peroxidase and catalase ascorbate and high ascorbic acid content and showed the lowest accumulation of hydrogen peroxide and lipid peroxidase, compared to a sensitive genotype that had the lowest activity of antioxidant enzymes and ascorbic acid content and the highest content of hydrogen peroxide and lipid peroxidase [7,8]. Reactive oxygen species (ROS) are generated in plants upon exposure to stressful conditions [9]. ROS are byproducts of numerous enzymatic reactions in various cell compartments [10].

The combating ROS in plants during stressful conditions is maintained by the enzymatic components comprising of the superoxide dismutase (SOD), ascorbate peroxidase (APX), guaiacol peroxidase (GPX), glutathione-S-transferase (GST), and catalase (CAT). The omnipresent nature of these enzymatic components underlies the necessity of the detoxification of ROS for cellular survival [9,10].

One of the most important goals of plant breeding is to produce new wheat cultivars with a high degree of drought tolerance. Thus, the first step is to select the potential germplasm that contains genotypic differences for drought tolerance [11].

Some authors [12] studied *Triticum* genotypes with three levels of polyploidy: hexaploid, tetraploid, and diploid, submitted to a stress of 4, 8, and 12 days, respectively. In general, catalase showed an increase or maintenance in the early stages of drought and then a decrease as the magnitude of stress increased. In contrast, peroxidase increased to water stress.

According to [13], who studied the role of the plant’s antioxidant system in stress tolerance, drought induced in two different stages after anthesis resulted in increased accumulation of oxygenated water and decreased ascorbic acid content. Antioxidant enzymes such as ascorbate peroxidase and catalase have increased under water stress conditions. A drought-tolerant genotype had the highest activity of ascorbate peroxidase and catalase and high ascorbic acid content and also showed the lowest accumulation of oxygenated water and lipid peroxidase. By comparison, a sensitive genotype had the lowest antioxidant enzyme activity and ascorbic acid content and the highest content of oxygenated water and lipid peroxidase.

A solution of polyethylene glycol (PEG) can be used to induce drought stress that is measured using a timescale of days after treating the seeds with the PEG solution. There are many different concentrations of PEG; therefore, it is essential to test a wide range of concentrations. In germination experiments using PEG, the seeds of genotypes are tested to different concentration [14,15].

Some authors [16] analysed five wheat cultivars submitted to 3, 6, and 9 days water stress, respectively. The activity of peroxidase and ascorbate peroxidase showed an initial increase. In cultivars that were found to be more stress-tolerant than others, peroxidase activity increased with increasing stress duration while ascorbate peroxidase activity decreased. The study conducted by [17] on an assortment of wheat cultivars and lines created at Șimnic regarding the phenol content and the activity of the antioxidants revealed that they are significantly influenced by genotype and environment. The Dropia variety was superior to the Boema variety and to the lines tested by point of view of phenol content.

Results reported by some authors suggest that water stress alters the balance between free radical production and enzymatic protection mechanisms in wheat plants [18]. Studies conducted by [19] investigated the effects of salicylic acid (SA) and cold on apoplastic protein levels and activities of apoplastic catalase (CAT), peroxidase (POX), and polyphenol oxidase (PPO) in winter wheat (*Triticum aestivum* cv. Dogu-88) leaves. When the activities with cold + SA treatment are compared to their cold treatments, CAT and POX activities were decreased while PPO activity was increased by SA.

The activities of antioxidant enzyme defence system depended on wheat cultivar, duration of drought, and the stage of leaf development [20].

Drought-tolerant genotypes also kept higher ascorbate compared with sensitive genotypes under stress and non-stress conditions, while peroxidase activity was not affected by drought stress [21]. Antioxidants and stress markers can be efficiently and economically used as biochemical indices to screen or to enrich wheat germoplasm for drought tolerance at the early seedling stage [21]. Plant materials should be phenotyped accurately using an appropriate assay and trait that has a direct relation to drought tolerance. Single-trait evaluation for drought tolerance to distinguish between tolerant and susceptible genotypes is not recommendable [22,23].

This study aimed at investigating the variability of some biochemical indicators (peroxidase, ascorbate peroxidase, and catalase) at the winter wheat assortments and identification of the sources showing a high antioxidant activity.

## 2. Results

In our experiment in south-western Oltenia, the activity of peroxidase increased as the stress induced by PEG treatment of different concentrations and at different doses increased. The varieties that showed an increase in the activity of the peroxidase are shown in Table 1.

The cultivars from the limited assortment were yellow highlighted (winter wheat cultivars tested for 14 years, year by year). It is observed that they are distributed everywhere in the range of the results obtained by the 50 cultivars, this fact indicating that the varieties that are part of this assortment and the obtained results could have a high probability of identifying the sources of stress tolerance for the south-western Oltenia drought conditions.

Among the varieties treated with PEG 10,000 25%, most of the Romanian varieties tested, components of the limited assortment, Gruia, Izvor, Lovrin 34, Boema, Delabrad, Glosa, Alex, Miranda, and Faur, presented super unitary values of the ratio between PEG/control treatment, which suggests drought tolerance.

Conversely, the activity of ascorbate peroxidase is lower in the tolerant varieties, so in Table 2, the varieties with subunit ratio were noted. Among them are the Izvor variety, known as the drought-tolerant variety, as well as other Romanian varieties: Alex, Delabrad, Lovrin 34, Miranda, Dor, and Romulus (Table 2).

Increased activity of catalase has been present in most varieties, so that the possibility of drought tolerance exists. Among the varieties listed in Table 3 are Romanian varieties: Dropia, Trivale, Nikifor, Simnic 30, Simnic 50, Faur, Glosa, Gruia, Miranda, and Flamura 85. Of the foreign varieties that maintain a high activity of catalase at higher dose stress intensification, we can see: Kristina, GH Hattyu, Karlygash, Esquisit, Lada, Enesco, GK Elet, Cubus, and GK Gobe (Table 3).

However, the correlation between YI in the limited assortment and the ratio of antioxidant enzyme content between PEG and control treatments does not exist, suggesting that none of these biochemical indicators represent a selection indicator for drought tolerance under the conditions of south-western Oltenia (Table 4).

From previous studies [24,25], it was clear that there is a close correlation between the YI index, which expresses drought tolerance, and the ratio between the stem length at PEG treatment 20% and the length of the stem at the control of 15 days from the sowing date (T1) or on average at three moments of determination (15, 24, and 35 days from sowing) and the ratio of the weight of the stem to PEG treatment 20% against the weight of the stem on the control, on the other hand.

In turn, these reports were correlated, as follows (Table 5):

The ratio of stem length to 20% PEG treatment and control on average of three determination times (Med) was correlated with:

- Ratio between peroxidase content after 25% PEG treatment and peroxidase content in the control-significant correlation.

The ratio of the stem weight to PEG treatment 20% and the weight of the stem to the control was correlated with:

- Ratio between catalase content at 25% PEG treatment and control catalase content—distinctly significant correlation;
- Ratio between catalase content at 40% PEG treatment and control catalase content—significant correlation.

Directly or indirectly, all these determinations mentioned above can be used as selection indicators for drought tolerance, and, depending on the results obtained from the varieties tested, certain parents may be suggested.

The biochemical indicators were correlated, as follows:

The ratio of peroxidase content to 25% PEG treatment to peroxidase content in the control was significantly correlated with:

1. The ratio between the stem length at 15% PEG treatment and the length of the stem at the control after 24 days from sowing (T2);
2. The ratio of stem length to 15% PEG treatment and control stem length on average on determinations made at 15, 24, and 35 days;
3. The ratio of stem length to 20% PEG treatment and control stem length on average on determinations made at 15, 24, and 35 days;
4. The ratio of stem weight to the 15% PEG treatment and the weight of the stem to the control.

When the PEG dose is increased to 40%, another correlation appears:

- Distinctly significant positive with the ratio of peroxidase content to 20% PEG treatment and peroxidase content to control.

The ratio of ascorbate peroxidase content to 25% PEG treatment and ascorbate peroxidase content control and ratio of ascorbate peroxidase to 40% PEG treatment and ascorbat peroxidase determined in control were significantly negatively correlated with reduction of seedling length in 40% PEG treatment compared to the control (treated with water).

## 3. Discussion

Drought (water deficit) one the emerging threat worldwide and adversely affects the morpho-physiology and biochemical activity of plants, finally leading to a decrease in the grain yield of wheat [26,27]. Additionally, [28] showed that drought stress is one of the main threats that negatively affected the morphological, physiological, and biochemical behaviours of plants than other abiotic stresses. Drought adversely deteriorated the plant metabolic process by affecting the photosynthesis and water relations of the plant and also the uptake of nutrients [29].

At the biochemical indices, there is a change of meaning of the classification because a high ratio of peroxidase and catalase but a lower ratio of ascorbate peroxidase are desirable. In our study, the best of this pattern is the folded Kristina cultivar. On the other hand, the Izvor cultivar, known for its drought tolerance, is not noticeable by very good values for the enzymatic reaction possibly involved in drought conditions. Our results suggest ways to improve the Izvor cultivar performance under water stress conditions by hybridizing it with Kristina or Dropia varieties. Thus, descendants can be selected that accumulate good growth in the presence of water stress (simulated by PEG treatment), with a better reaction of the enzyme apparatus.

The results of many authors suggest that ascorbate peroxidase, a central enzyme for ROS scavenging in plants, can be induced under abiotic and biotic stresses [30,31,32,33,34]. Thus, the antioxidant enzymes APX, SOD, POD, and CAT are produced under different environmental stresses (such as drought, salt, etc.) for scavenging the activity of ROS in plants [35,36].

Additionally, the peroxidase activity has intensified as stress induced by PEG treatment of different concentrations and in different doses increased. Among the varieties treated with PEG 10,000 25%, most of the Romanian varieties tested presented super unit values of the PEG/control ratio, suggesting tolerance to drought. In reverse, the activity of ascorbate peroxidase is lower in tolerant varieties. Among the varieties evidenced by the increased activity of catalase were the Romanian varieties: Dropia, Trivale, Nikifor, Simnic 30, Simnic 50, Faur, Glosa, Gruia, Miranda, and Flamura 85. Among the foreign varieties that have maintained a high catase activity to increase stress through a higher dose of PEG were Kristina, GH Hattyu, Karlygash, Esquisit, Lada, Enesco, GK Elet, Cubus, and GK Gobe.

Drought stress can occur at any growth stage and depends on the local environment. According to [11], genotypes may be tested for their drought tolerance at relevant and often different growth stages because some genotypes may tolerate drought at the germination or seedling stage, but these may be very sensitive to drought at the flowering stage or vice versa. The ability of seeds and young seedlings to cope with oxidative stress during early vegetative growth and biotic (attachment of the soil and seed-borne pathogens) and abiotic stresses (drought, salinity, heat, and chilling) is vital for crop performance and production [37]. High activities of antioxidant enzymes such as superoxide dismutase (SOD), catalase (CAT), peroxidase (POD), and ascorbate peroxidase (APX) have been recorded during seed germination, early growth, and biotic and abiotic stresses [38,39,40]. APX plays a considerable role in wheat drought tolerance by detoxifying plants from the accumulation of H_2_O_2_ [41]

There are many studies that suggest that the yield index is correlated to antioxidant activity [38,39,40,41,42]. Generally, the genotypes respond differently to drought tolerance at different growth stages [11]. Some wheat genotypes exhibited a similar pattern of stress response, comprising proline accumulation, rise in hydrogen peroxide content, oxidative damage to membrane lipids, and increase in total antioxidant and antiradical activities, phenolic and flavonoid content, ascorbate and glutathione pools, and mobilization of superoxide dismutase (SOD), catalase (CAT), and peroxidase (POX) enzyme isoforms [42]. Other results reveal the important role of certain chloroplast chaperone proteins in drought stress response and different strategies of stress adaptation depending on the wheat genotype [43]. According to [44], the combination of lower N supply and water deprivation (osmotic stress induced by polyethylene glycol treatment) led to greater damage of the photosynthetic efficiency and a higher degree of oxidative stress than the individually applied stresses. Plant materials should be phenotyped accurately using an appropriate assay and trait that has a direct relation to drought tolerance [22].

Reactive oxygen species (ROS) plays an important signalling role in plants, controlling processes such as growth, development, and especially response to biotic and abiotic environmental stressors [45,46]. However, ROS are unable to cause damage, as they are being scavenged by different antioxidant mechanisms [47,48,49].

Plants treated with herbicides, similarly to those grown under various abiotic stress conditions, are subjected to enhanced attacks by ROS. According to [50], the stress markers, enzymatic and non-enzymatic antioxidant defence, were additionally increased during the stress period after the combined herbicide and drought treatment.

## 4. Materials and Methods

The experiments were located in the South-West area of Oltenia region (Romania). This belongs to the temperate climate zone, with Mediterranean influences due to its south-western position. The position and the depressional feature of the land it occupies, close to the curvature of the Carpathian–Balkan mountain range, determine, on the whole, a warmer climate than in the central and northern part of the country, with an annual average of 10–11.5 °C.

### 4.1. Plant Materials

Fifty wheat varieties of various origins were tested in the laboratory to detect differences between biochemical indicators: peroxidase, ascorbate peroxidase, and catalase. Fresh tissue, necessary for enzymatic analysis, was collected after 30 days in which the seedlings were grown in a controlled environment, under three experimental conditions (H_2_O, 25% PEG 10,000, and 40% PEG 4000).

From 50 varieties, 13 varieties were also field tested for the period 2002–2015, and the YI-specific drought tolerance index was calculated from yield data from the field, taking into account the average yield of years with the most severe drought 2002 and 2003 as Ys. This is the limited assortment.

Field experiments were placed in triple balanced grid without repeating the basic scheme.

### 4.2. Laboratory Researche Methods

Plants can be protected by antioxidant synthesis and by increasing the activity of antioxidant enzymes (peroxidase, superoxide dismutase, and catalase). The response of plants to exposure to water stress can be determined by different mechanisms, including the ability to maintain high levels of antioxidants and to regenerate them.

Peroxidase is the most extensively used enzyme as a biochemical marker of plant growth and development processes. The implications of peroxidases in plant physiology are multiple, but the most intensively studied refer to participation in the control of cell growth.

The laboratory analyses were carried out in 2017. The soil used in the planting pots had the same origin and was subsequently dried and brought to a uniform humidity.

Fifty variants were sown in plant pots containing the same amount of soil. After sprouting, six seedlings were kept in each vegetation pot. These were placed in the Sanyo growth chamber, previously adjusted to the optimal temperature, light, and atmospheric humidity parameters for proper growth of wheat plants (Table 6).

The enzymatic determinations were performed according to the working methodology specific to the biochemical analyses. Fresh tissue was homogenized with 0.1 M phosphate buffer (pH = 7.0) containing 0.1 mM ascorbic acid and 0.1 mM EDTA. The homogenate was centrifuged 20 min at 10,000 r.p.m. (rotation per minute), and the supernatant was used for enzyme assays.

- The activity of peroxidase (guaiacol-peroxidase type E.C.1.11.1.7) was determined colorimetrically at λ = 470 nm and was expressed as the variation in absorbance per minute due to the oxidation of guaiacol from the extract of one gram of fresh substance [51].
- The activity of catalase (E.C.1.11.1.6) was determined by the Sinha method by colorimetrically determining the amount of H_2_O_2_ decomposed for 1 min by the enzyme in 1 g fresh substance. The method is based on the fact that potassium chromate in acidic medium is reduced by hydrogen peroxide to chromic acetate, which can be colorimetric at 570 nm [52,53].

### 4.3. Statistical Analysis

The paper contains the YI computation and the calculation of correlation coefficients.

Yield index [54],

$$\mathrm{YI}=\frac{Ys}{\bar{Y}s}$$

where *Ys* is the production in the dry year and *Ȳs* is the average of the production in the dry year, best reflects the behaviour under stress conditions, compared to the average of all varieties. It is not influenced by other conditions and therefore seems the most adequate to characterize the ability of indirect methods to describe the drought resistance. The correlations between yield index and the antioxidant activity for the limited assortment were calculated.

The correlations were performed with the Pearson correlation test after [55].

## 5. Conclusions

Peroxidase activity has intensified as stress induced by PEG treatment of different concentrations and in different doses increased. Among the varieties treated with PEG 10,000 25%, most of the Romanian varieties tested (Gruia, Izvor, Lovrin 34, Boema, Delabrad, Glosa, Alex, Miranda, and Faur) presented super unit values of the PEG/control ratio, suggesting tolerance to drought. In reverse, the activity of ascorbate peroxidase is lower in tolerant varieties. The varieties with a subunit report were remarked upon. Among them are the Izvor variety, known as the drought-tolerant variety, as well as other Romanian varieties: Alex, Delabrad, Lovrin 34, Miranda, Dor, and Romulus.

Among the varieties evidenced by the increased activity of catalase were the Romanian varieties: Dropia, Trivale, Nikifor, Simnic 30, Simnic 50, Faur, Glosa, Gruia, Miranda, and Flamura 85. Among the foreign varieties that have maintained a high catalase activity to increase stress through a higher dose of PEG were Kristina, GH Hattyu, Karlygash, Esquisit, Lada, Enesco, GK Elet, Cubus, and GK Gobe.

There was no significant correlation between field behaviour to stress, expressed by the YI index on the limited assortment and ratios of antioxidant enzyme content between PEG and control treatments, suggesting that none of these biochemical indicators individually represent a selection marker for drought tolerance under the conditions of south-western Oltenia.

From the improver’s point of view, thus, given that the Izvor cultivar does not stand out for very good values for the enzyme (activity of catalase—1814 ratio PEG 10,000 25%/control and 1099 ratio PEG 4000 40%/control) device possibly involved in drought behaviour, one can expect that from its hybridization with the Kristina (activity of catalase —3519 ratio PEG 10,000 25%/control and 6171 ratio PEG 4000 40%/control) or Dropia (activity of catalase—4721 ratio PEG 10,000 25%/control and 5.007 ratio PEG 4000 40%/control cultivars), it is possible to select progeny that will cumulate good growth in the presence of water stress (simulated by PEG treatment), causing a better device enzymatic reaction.

## Figures and Tables

**Table 1:** The activity of peroxidase in an assortment of 50 wheat cultivars analysed.

No.	Cultivar	PEROXIDASE (ΔA/1 min/1 gsp)				
		In Normal Conditions (Water–Control)	In Water-Stress-Induced Conditions (25% PEG 10,000)	In Water-Stress-Induced Conditions (40% PEG 4000)	Ratio PEG (25%/Control)	Ratio PEG (40%/Control)
35.	KRISTINA	57.60	211.43	319.02	3.671	5.539
39.	MOLDAU	91.69	308.8	0	3.368	0.000
40.	MV PALMA	66.21	220.78	0	3.335	0.000
50.	TRIVALE	23.27	71.31	0	3.064	0.000
31.	GK HATTYU	104.88	293	431.14	2.794	4.111
36.	LADA	72.94	188.09	141.2	2.579	1.936
48.	SHOHAM	71.46	177.44	0	2.483	0.000
30.	GRUIA	75.75	176.8	200.6	2.334	2.648
47.	ROMULUS	75.16	168.75	0	2.245	0.000
34.	KARLYGASH	72.01	158.66	275.62	2.203	3.828
41.	NIKIFOR	85.89	180.38	100.54	2.100	1.171
29.	GK GOBE	119.08	238.71	174.45	2.005	1.465
43.	ORQUAL	69.33	134.46	0	1.939	0.000
25.	GIAVA	111.83	216	321.96	1.932	2.879
33.	IZVOR	114.15	216.16	190.17	1.894	1.666
27.	GK ELET	105.89	199.7	387	1.886	3.655
21.	EXOTIC	76.19	141	332	1.851	4.358
42.	ORATORIO	72.12	128.97	0	1.788	0.000
18.	ELIANA	85.96	125.55	87.59	1.461	1.019
26.	GK DAVID	207.61	301	543.27	1.450	2.617
46.	ROMANSA	111.45	157.97	76.76	1.417	0.689
11.	CUBUS	108.57	151.5	219.64	1.395	2.023
1.	AGRON	134.4	176	218.3	1.310	1.624
5.	BITOP	140.21	176.25	162.7	1.257	1.160
38.	LOVRIN 34	126.92	144.34	0	1.137	0.000
6.	BOEMA	151.28	171.53	165	1.134	1.091
13.	DELABRAD	122.7	137.88	166.65	1.124	1.358
28.	GLOSA	137.53	151.6	372	1.102	2.705
2.	ALEX	130.9	141.28	191.75	1.079	1.465
23.	FLAMURA 85	171	176	316	1.029	1.848
3.	AZTEC	138	140.77	161	1.020	1.167
32.	MIRANDA	90.6	92.25	170.25	1.018	1.879
22.	FAUR	107.55	108	184.48	1.004	1.715
14.	DEMETRA	129.66	128.76	215.12	0.993	1.659
7.	SIMNIC 50	121.1	113.92	255.1	0.941	2.107
16.	DROPIA	120.4	111.57	140.68	0.927	1.168
19.	ENESCO	132.89	121.96	321.83	0.918	2.422
24.	GABRIELA	149.67	135.62	265	0.906	1.771
20.	ESQUISIT	199.74	168.06	219.21	0.841	1.097
12.	DARIEL	132.97	110.44	163.74	0.831	1.231
4.	BEZOSTAIA	162.83	130.92	175	0.804	1.075
15.	DOR	160.66	126.42	166.11	0.787	1.034
17.	DUNAI	164.75	125.78	211.99	0.763	1.287
49.	SIMNIC 30	59.58	43.96	0	0.738	0.000
10.	CRINA	133.22	84.7	138.87	0.636	1.042
44.	JULIUS	207.74	121.65	0	0.586	0.000
8.	CAPO	179.5	103.76	182.28	0.578	1.015
37.	LITERA	139.04	77.74	0	0.559	0.000
45.	NATHAN	238.33	113.04	0	0.474	0.000
9.	CAROLINA	126.33	56.63	163.25	0.448	1.292

**Table 2:** The activity of ascorbat peroxidase in an assortment of 50 wheat cultivars analysed.

No.	Cultivar	ASCORBAT PEROXIDAZE (μgAsA/1 min/1 gsp)				
		In Normal Conditions—Water–Control	In Water-Stress-Induced Conditions—25% PEG 10,000	In Water-Stress-Induced Conditions—40% PEG 4000	Ratio PEG 25%/Control	Ratio PEG 40%/Control
50.	TRIVALE	4693	40,522	0	8.635	0.000
49.	SIMNIC 30	4754	35,528	0	7.473	0.000
29.	GK GOBE	2990	21,994	3661	7.356	1.224
8.	CAPO	16,313	57,206	4550	3.507	0.279
9.	CAROLINA	5834	17,921	11,101	3.072	1.903
7.	SIMNIC 50	9829	29,888	14,349	3.041	1.460
11.	CUBUS	4606	11,865	13,154	2.576	2.856
40.	MV PALMA	52,089	129,907	0	2.494	0.000
10.	CRINA	13,795	33,881	30,699	2.456	2.225
41.	NIKIFOR	75,225	179,104	41,893	2.381	0.557
37.	LITERA	26,825	62,474	0	2.329	0.000
14.	DEMETRA	9350	20,147	6711	2.155	0.718
48.	SHOHAM	34,285	70,888	0	2.068	0.000
23.	FLAMURA 85	31,788	60,423	80,790	1.901	2.542
5.	BITOP	13,523	24,456	20,302	1.808	1.501
6.	BOEMA	11,930	20,898	0	1.752	0.000
22.	FAUR	29,801	51,107	34,830	1.715	1.169
24.	GABRIELA	33,376	51,546	43,200	1.544	1.294
30.	GRUIA	18,476	28,177	13,396	1.525	0.725
21.	EXOTIC	20,906	31,348	52,677	1.499	2.520
3.	AZTEC	12,420	17,802	4565	1.433	0.368
28.	GLOSA	28,813	37,094	14,970	1.287	0.520
39.	MOLDAU	63,595	70,754	0	1.113	0.000
43.	ORQUAL	35,074	38,866	0	1.108	0.000
16.	DROPIA	23,299	25,689	22,446	1.103	0.963
17.	DUNAI	28,531	31,389	18,868	1.100	0.661
36.	LADA	13,827	13,470	16,152	0.974	1.168
12.	DARIEL	7299	6995	12,564	0.958	1.721
27.	GK ELET	25,027	23,328	7278	0.932	0.291
2.	ALEX	9663	9001	5758	0.931	0.596
25.	GIAVA	56,518	50,899	61,391	0.901	1.086
45.	NATHAN	34,463	29,747	0	0.863	0.000
13.	DELABRAD	20,398	17,253	20,925	0.846	1.026
15.	DOR	20,618	17,412	39,525	0.845	1.917
35.	KRISTINA	37,092	29,585	0	0.798	0.000
47.	ROMULUS	46,875	35,744	0	0.763	0.000
38.	LOVRIN 34	29,747	21,739	0	0.731	0.000
33.	IZVOR	43,547	29,441	26,106	0.676	0.599
1.	AGRON	10,847	6428	6007	0.593	0.554
46.	PKB ROMANSA	70,721	39,318	60,942	0.556	0.862
32.	MIRANDA	51,011	27,675	15,796	0543	0.310
26.	GK DAVID	35,090	18,654	0	0.532	0.000
4.	BEZOSTAIA	9221	45,85	5098	0.497	0.553
42.	ORATORIO	73,625	31,888	0	0.433	0.000
44.	JULIUS	80,042	30,550	0	0.382	0.000
19.	ENESCO	65,789	24,314	12,227	0.370	0.186
18.	ELIANA	36,772	13,413	29,207	0.365	0.794
31.	GK HATTYU	17,537	6002	12,975	0.342	0.740
34.	KARLYGASH	34,110	11,376	6852	0.334	0.201
20.	ESQUISIT	10,981	2838	20,284	0.258	1.847

**Table 3:** The activity of catalase in an assortment of 50 wheat cultivars.

No.	Cultivar	CATALASE				
		In Normal Conditions—Water–Control	In Water-Stress-Induced Conditions—25% PEG 10,000	In Water-Stress-Induced Conditions—40% PEG 4000	Ratio PEG 25%/Control	Ratio PEG 40%/Control
48.	SHOHAM	656	3318	-	5.058	-
16.	DROPIA	298	1407	1492	4.721	5.007
35.	KRISTINA	896.8	3155.81	5534.08	3.519	6.171
4.	BEZOSTAIA	1536.98	4471	1087.7	2.909	0.708
50.	TRIVALE	987	2765	-	2.801	-
42.	ORATORIO	1652	4578	-	2.771	-
31.	GK HATTYU	1945.44	5378.13	5536.33	2.764	2.846
18.	ELIANA	1046	2862	290	2.736	0.277
41.	NIKIFOR	1288	3422	3422	2.657	2.657
9.	CAROLINA	435.73	1142.05	-	2.621	-
34.	KARLYGASH	1819.21	3519.15	2557.91	1.934	1.406
49.	SIMNIC 30	982	1876	-	1.910	-
20.	ESQUISIT	1749	3309	3137	1.892	1.794
36.	LADA	1482.57	2753.87	4020.1	1.857	2.712
7.	SIMNIC 50	366.97	680.13	-	1.853	-
33.	IZVOR	929	1685.36	1021.05	1.814	1.099
22.	FAUR	763	1362.8	-	1.786	-
46.	PKB ROMANSA	1128	1864	-	1.652	-
19.	ENESCO	701	1141	4565	1.628	6.512
28.	GLOSA	1782.55	2868.62	3832.3	1.609	2.150
27.	GK ELET	1993.32	3110.42	4885.5	1.560	2.451
43.	ORQUAL	1808	2784	-	1.540	-
30.	GRUIA	1839.45	2604	2703.61	1.416	1.470
11.	CUBUS	1253	1654	1497	1.320	1.195
45.	NATHAN	1387	1829	-	1.319	-
32.	MIRANDA	1432.37	1668.02	1904.12	1.165	1.329
26.	GK DAVID	3992.5	4158.68	1923.07	1.042	0.482
29.	GK GOBE	3472.56	3501.76	3645.2	1.008	1.050
12.	DARIEL	1370	1380	965	1.007	0.704
39.	MOLDAU	1208	1211	-	1.002	-
23.	FLAMURA 85	5086	5086	5086	1.000	1.000
24.	GABRIELA	5086	5086	5086	1.000	1.000
21.	EXOTIC	1115	1103.4	1274	0.990	1.143
2.	ALEX	1020.46	921.75	3071.01	0.903	3.009
40.	MV PALMA	2200	1877	-	0.853	-
15.	DOR	1173	990	1265	0.844	1.078
8.	CAPO	1020	854.3	-	0.838	-
44.	JULIUS	2100	1685	-	0.802	-
38.	LOVRIN 34	1625	1265	-	0.778	-
1.	AGRON	3010	2285.71	4605.66	0.759	1.530
3.	AZTEC	2700	1898.88	2207.8	0.703	0.818
37.	LITERA	987	674	-	0.683	-
10.	CRINA	820.55	542.1	382.04	0.661	0.466
13.	DELABRAD	1450	938	-	0.647	-
5.	BITOP	3300.23	2086.96	1082.78	0.632	0.328
47.	ROMULUS	4562	2647	-	0.580	-
25.	GIAVA	3014.31	1266.82	3274.2	0.420	1.086
14.	DEMETRA	1662	286	1577	0.172	0.949
6.	BOEMA	3800	334.38	-	0.088	-
17.	DUNAI	3570	312	1342	0.087	0.376

**Table 4:** The correlation between the YI index and the antioxidant activity for the limited assortment.

Cultivar	YI	Ratio PEROX PEG 25%/ct	Ratio PEROX PEG 40%/ct	Ratio ASC PEG 25%/ct	Ratio ASC PEG 40%/ct	Ratio CAT PEG 25%/ct	Ratio CAT PEG 40%/ct
GLOSA	1.252	1.102	2.705	1.287	0.520	1.609	2.150
GRUIA	1.220	2.334	2.648	1.525	0.725	1.416	1.470
IZVOR	1.219	1.894	1.666	0.676	0.599	1.814	10.991
FAUR	1.153	1.004	1.715	1.715	1.169	1.786	0.000
DELABRAD	1.116	1.124	1.358	0.846	1.026	0.647	0.000
CRINA	0.996	0.636	1.042	2.456	2.225	0.000	0.000
ALEX	0.955	1.079	1.465	0.931	0.596	0.903	3.009
DROPIA	0.941	0.927	1.168	1.103	0.963	4.721	5.007
SIMNIC 30	0.895	0.738	1.029	7.473	5.972	1.910	-
BEZOSTAIA	0.854	0.804	1.075	0.497	0.553	2.909	0.708
BOEMA	0.847	1.134	1.091	1.752	1.400	0.000	0.000
ROMULUS	0.800	2.245	3.130	0.763	0.609	0.580	0.000
LOVRIN 34	0.753	1.137	1.585	0.731	0.584	0.000	0.000
Corelation with YI		0.25	0.31	−0.11	−0.18	0.14	0.42

**Table 5:** Correlations between the characters determined in south-western Oltenia on a limited assortment of wheat varieties.

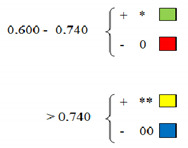		**Ratio Length Stem PEG 15%/ct T_1_**	**Ratio Length Stem PEG 15%/ct T_2_**	**Ratio Length Stem PEG 15%/ct T_3_**	**Ratio Length Stem PEG 15%/ct aver.**	**Ratio Length Stem PEG 20%/ct T_1_**	**Ratio Length Stem PEG 20%/ct T_2_**	**Ratio Length Stem PEG 20%/ct T_3_**	**Ratio Length Stem PEG 20%/ct Aver.**	**Ratio Weight Root PEG 15%/ct**	**Ratio Weight Root PEG 20%/ct**	**Ratio Weight Stem PEG 15%/ct**	**Ratio Weight Stem PEG 20%/ct**	**Ratio Root/Stem PEG 15%/Root/Stem/ct**	**Ratio Root/Stem PEG 20%/Root/Stem/ct**	**Seed Germ. Stress/Normal Var (%)**
		**1**	**2**	**3**	**4**	**5**	**6**	**7**	**8**	**9**	**10**	**11**	**12**	**13**	**14**	**15**
Peroxidase PEG 25%/per ct	23	0.491	0.727	0.380	0.710	0.469	0.589	0.296	0.636	0.540	0.033	0.702	0.150	0.095	−0.131	0.224
Peroxidase PEG 40%/per ct	24	0.463	0.668	0.412	0.684	0.321	0.479	0.322	0.515	0.436	−0.114	0.689	−0.081	0.109	−0.001	0.269
Asc PEG 25%/asc ct	25	−0.090	−0.383	0.388	−0.062	−0.108	−0.055	0.208	0.002	−0.186	0.076	−0.223	−0.248	−0.007	0.402	−0.407
Asc PEG 40%/asc ct	26	−0.231	−0.440	0.379	−0.173	−0.205	−0.095	0.219	−0.066	−0.220	0.091	−0.242	−0.231	−0.042	0.384	−0.467
Cat PEG 25%/cat ct	27	0.169	0.352	0.127	0.280	0.183	0.740	0.357	0.561	0.250	0.796	0.223	0.762	−0.105	0.204	−0.406
Cat PEG 40%/cat ct	28	0.279	0.326	0.003	0.294	0.236	0.656	0.170	0.479	0.218	0.734	0.181	0.700	−0.084	0.189	−0.259
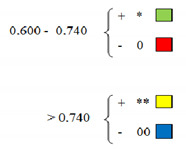		**Red. Coleoptil Length (%)**	**Ratio Length pl PEG 25%/Length pl. ct**	**Ratio Length pl PEG 40%/Length pl. ct**	**Initial Water Cont. (%)**	**Water Loss after 4 h (%)**	**Water Loss after 20 h (%)**	**Water Loss after 24 h (%)**	**Peroxidase PEG 25%/per ct**	**Peroxidase PEG 40%/per ct**	**Asc per PEG 25%/Asc per ct**	**Asc per PEG 40%/Asc per ct**	**Catalase PEG 25%/Cat ct**	**Catalase PEG 40%/Cat ct**		
		**16**	**17**	**18**	**19**	**20**	**21**	**22**	**23**	**24**	**25**	**26**	**27**	**28**		
Peroxidase PEG 25%/per ct	23	−0.393	0.045	0.111	0.204	0.590	−0.295	0.114	1							
Peroxidase PEG 40%/per ct	24	−0.781	0.128	0.115	0.067	0.156	−0.351	−0.194	0.771	1						
Ascorbat peroxidase PEG 25%/asc ct	25	0.235	−0.468	−0.863	−0.055	0.156	−0.200	−0.131	−0.336	−0.298	1					
Ascorbat peroxidase PEG 40%/asc ct	26	0.338	−0.450	−0.843	−0.043	0.027	−0.127	−0.088	−0.397	−0.404	0.985	1				
Catalase PEG 25%/cat ct	27	0.202	−0.255	0.049	0.242	0.410	0.009	0.255	0.290	−0.042	−0.147	−0.130	1			
Catalase PEG 40%/cat ct	28	0.109	−0.115	0.160	0.189	0.446	0.006	0.274	0.289	0.005	−0.258	−0.260	0.932	1		

**Table 6:** Optimal parameters for proper growth of wheat plants.

Parameters	Values									
Time (h)	0:00	3:00	6:00	9:00	11:00	13:00	15:00	17:00	19:00	22:00
Temperature (°C)	15.0	14.0	15.0	18.0	20.0	25.0	25.0	20.0	18.0	16.0
Light (lux)	0	0	1	2	4	5	4	3	1	0
Humidity (%)	60	65	65	60	55	55	55	55	55	55

## Data Availability

All the data supporting this article were included in the main text.
